# BL-MOL-AR Project, Preliminary Results about Liquid Biopsy: Molecular Approach Experience and Research Activity in Oncological Settings

**DOI:** 10.1055/s-0043-1771193

**Published:** 2023-07-14

**Authors:** Alessandro Pancrazzi, Francesco Bloise, Alice Moncada, Roberta Perticucci, Stefania Vecchietti, Francesca Pompili, Francesca Ricciarini, Silvia Lenzi, Cristina Gatteschi, Sabrina Giusti, Maria Pia Rosito, Sabrina Del Buono, Paola Belardi, Alessandra Bruni, Filippo Borri, Andrea Campione, Lorella Laurini, Rossella Occhini, Loretta Presenti, Viviana Viticchi, Maja Rossi, Sara Bardi, Antonio D'Urso, Simona Dei, Duccio Venezia, Raffaele Scala, Carmelo Bengala, Nicola Libertà Decarli, Andrea Carnevali, Carlo Milandri, Agostino Ognibene

**Affiliations:** 1 Laboratory Medicine Department, Clinical and Molecular Pathology Sector, San Donato Hospital, Arezzo, Italy; 2 Oncology Department, Unit of Medical Oncology, San Donato Hospital, Arezzo, Italy; 3 Oncology Department, Pathological Anatomy Laboratory, San Donato Hospital, Italy; 4 Laboratory Medicine Department, Clinical and Molecular Pathology Sector, Misericordia Hospital, Grosseto, Italy; 5 General Management, Local Health Unit South-East Tuscany, Tuscany, Italy; 6 Diagnostic Imaging Department, Radiology Unit, San Donato Hospital, Arezzo, Italy; 7 Cardio Thoracic Neuro Vascular Department, Pneumology Unit, San Donato Hospital, Arezzo, Italy; 8 Oncology Department, Unit of Medical Oncology, Misericordia Hospital, Grosseto, Italy; 9 Oncology Department, Pathological Anatomy Laboratory, Misericordia Hospital, Grosseto, Italy

**Keywords:** liquid biopsy, cfDNA, NGS

## Abstract

**Background** Liquid biopsy is mainly used to identify tumor cells in pulmonary neoplasms. It is more often used in research than in clinical practice. The BL-MOL-AR study aims to investigate the efficacy of next-generation sequencing (NGS) and clinical interpretation of the circulating free DNA (cfDNA) levels. This study reports the preliminary results from the first samples analyzed from patients affected by various neoplasms: lung, intestinal, mammary, gastric, biliary, and cutaneous.

**Methods** The Biopsia Liquida-Molecolare-Arezzo study aims to enroll cancer patients affected by various malignancies, including pulmonary, intestinal, advanced urothelial, biliary, breast, cutaneous, and gastric malignancies. Thirty-nine patients were included in this preliminary report.

At time zero, a liquid biopsy is executed, and two types of NGS panels are performed, comprising 17 genes in panel 1, which is already used in the routine tissue setting, and 52 genes in panel 2. From the 7th month after enrollment, 10 sequential liquid biopsies are performed up to the 17th month. The variant allele frequency (%) and cfDNA levels (ng/mL) are measured in every plasmatic sample.

**Results** The NGS results obtained by different panels are similar even though the number of mutations is more concordant for lung pathologies. There are no significant differences in the actionability levels of the identified variants. Most of the molecular profiles of liquid biopsies reflect tissue data.

**Conclusions** Preliminary data from this study confirm the need to clarify the limitations and potential of liquid biopsy beyond the lung setting. Overall, parameters related to cfDNA levels and variant allele frequency could provide important indications for prognosis and disease monitoring.

## Background

The improvement of diagnostic techniques and progression monitoring of solid tumors are today the most important challenges in oncological research. In recent decades, the focus of the scientific community has been on diagnostic tools considering the critical issues in practices such as tissue biopsy. 1 2 It is now an established concept that reduction of mortality in cancer patients can be partly achieved by an analysis of samples more representative of the tumor genotype obtained with less invasiveness than tissue biopsy practices. 3 4 5 Investigation targets of liquid biopsy such as circulating tumor cells (CTCs), circulating tumor deoxyribonucleic acid (ctDNA), proteins, metabolites, exosomes, messenger ribonucleic acids (RNAs), and microRNAs are increasingly being studied in the translational setting. 6 7 8 9 10 11 12 13 14 15

The gold standard for the study of tumor profile is tissue biopsy, but this technique presents a highly debated series of critical issues. In general, tissue biopsy is invasive, risky, and not easy to perform for specific anatomical locations. Another critical aspect is the clonal heterogeneity of the tumor. Tumors are characterized by various cellular subpopulations presenting various alterations. Furthermore, over time, due to the nature of the neoplasm and the iatrogenic effect of therapy, these cells undergo genetic and epigenetic variations. This scenario increases the possibility of discrepancies between primary and metastatic lesions, making it difficult to monitor and evaluate response to adequate therapy. 16 17

The appropriateness of liquid biopsy and extension of this method to various oncological settings are still hotly debated. Various current guidelines suggest the use of liquid biopsy in all cases in which obtaining a tissue sample is not possible (a more frequent event in the pulmonary setting), but it is also true that this technique is not yet codified and widely used, and very little data are available in the literature. 17 18 19

## Methods

### Patients and Design of the Study

The multicenter study BL-MOL-AR was approved by the Regional Ethics Committee for Clinical Trials of the Tuscany Region (Protocol no.: 2021/000310). The patients were recruited by the Oncology Department of the South East Tuscany Local Health Unit at the hospitals of San Donato in Arezzo and Misericordia in Grosseto.

The two participating centers, Clinical Molecular Pathology sectors of the Analysis Laboratory and Pathological Anatomy, investigated the biological samples. Clinical Molecular Pathology sectors performed molecular examinations on plasma samples and tissue material, whereas pathological anatomy processed the tissue material in terms of embedding and section selection. The BL-MOL-AR study aims to evaluate the validity of the use of liquid biopsy in the personalized therapy setting of choice in cancer patients (melanoma, colon, lung, breast, gastric cancer, cholangiocarcinoma, and advanced urothelial cancer). In particular, it is proposed to evaluate the validity of molecular assays on ctDNA through investigation methods such as next-generation sequencing (NGS) and digital droplet polymerase chain reaction (ddPCR).

The study involves selecting patients in the diagnostic and therapeutic frameworks for melanoma; colon, lung, breast, and gastric cancer; cholangiocarcinoma; and advanced urothelial cancer.

We expected to enroll a total of at least 100 to 120 patients for the study. These patients were selected by oncologists and after obtaining their informed consent, they were to be included in a monitoring program that will use the conventional methods recognized by the respective guidelines and the molecular investigation techniques applied for the analysis of ctDNA.

Patients included the study must be younger than 80 years and must have one of the pathological conditions or be in the following clinical stages:

- Diagnosis, in naive patients with advanced lung cancer, in the cases where standard lung biopsies have failed to provide an adequate quantity/quality of material for molecular analysis.
- At the time of disease progression to epidermal growth factor receptor (EGFR) tyrosine kinase inhibitors (EGFR-TKIs) in patients with advanced non-small-cell lung cancer (NSCLC).
- In the monitoring/staging/diagnosis of neoplastic lung or intestinal or skin disease (melanoma).
- In cases of diagnosis of estrogen receptor positive (ER + ) and negative human epidermal growth factor receptor 2 (HER2–) breast cancer.
- At the time of diagnosis or progression of cases of gastric cancer.
- At the time of diagnosis or progression of cases of cholangiocarcinoma.
- In clinical cases of metastatic urothelium carcinoma.

In the enrolled patients, after the first therapeutic/chemotherapy cycle, in the post-therapeutic staging phase, at the same time as complete radiological monitoring with program variation of more or less 3 days after radiological investigations or in any case starting from the 7th month from time zero (T0), monthly liquid biopsy samples are taken up to a total of 10 months of monitoring, that is, the 17th month from T0. It should be noted that venous blood sampling is always provided for oncological patients for routine investigations for clinical use; during these checks, liquid biopsy envisaged by the protocol in question can be done simultaneously.

Molecular tests on ctDNA extracted at T0 were done using the NGS technology by applying two distinct panels, one for diagnostic setting, CE-IVD (European Conformity, In Vitro Diagnostics) marked, targeting 17 genes (henceforth denominated panel 1) and another for research use only (RUO) consisting of 52 (44 for DNA targeting) target genes, which is henceforth referred to as panel 2 (see NGS material and methods section for specific details).

**Fig. 1:**
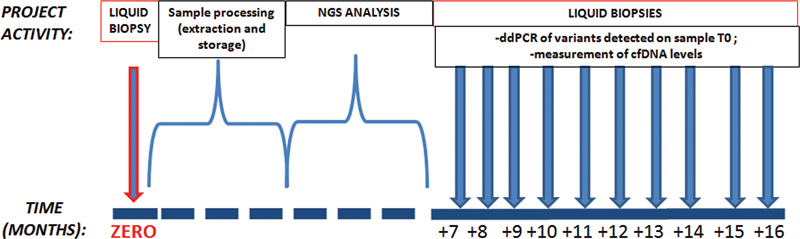
Workflow concerning the analysis of cfDNA on enrolled patients. The NGS analysis, performed with Panels 1 and 2, is executed on the circulating DNA of the first sampling at time zero (T0). The sequential collecting starts from the seventh month to monitor the variants of clinical significance found in the NGS analysis. The quantification of cfDNA is performed on all liquid biopsies. cfDNA, circulating free DNA; ddPCR, droplet digital polymerase chain reaction; NGS, next-generation sequencing.

The cfDNA levels of each liquid biopsy sample collected in the study were also measured. In pathogen-negative patients, probably pathogenic patients, or those with variants of uncertain significance (VUS), fluctuation of cfDNA levels was analyzed as the only laboratory monitoring parameter ( Fig. 1 ).

In the cases where it is possible to perform NGS analyses through CE-IVD panel on tissue material (biopsies or surgical pieces), a method normally used in diagnostic routine, these molecular results will be compared with those obtained through the same NGS panel on the plasma of the liquid biopsies performed in the study.

### Patients

**Fig. 2:**
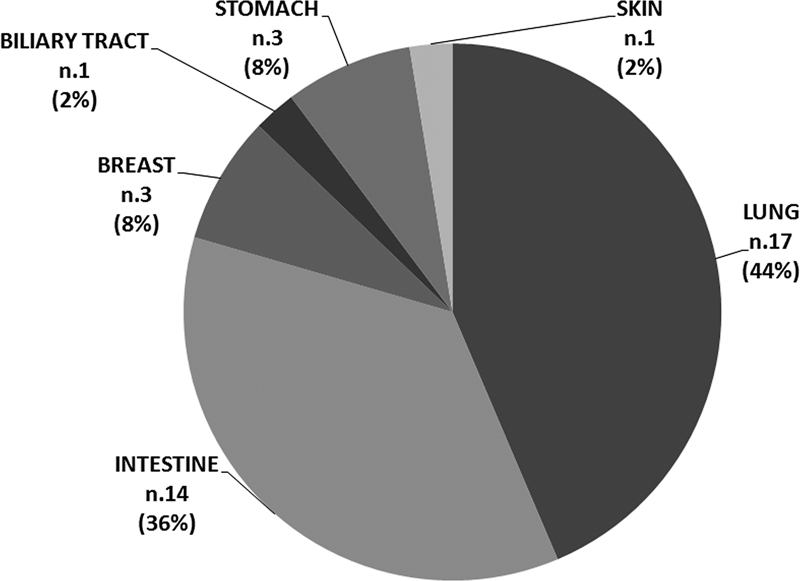
Distribution of tumor origin in the study population.

In this preliminary phase of the study, 39 patients were analyzed, and the study population is distributed according to the neoplastic pathology diagnosis as follows: 14 intestinal metastatic (colon), 17 lung metastatic, 3 gastric, 3 breasts, 1 biliary tract (cholangiocarcinoma), and 1 skin (melanoma; Fig. 2 ). Informed written consent was obtained from all the patients who participated in the study.

### DNA Extraction, Purification, and Quality Estimation Methods

All peripheral blood samples in the study were taken by appointment by expert staff. To reduce in vitro hemolysis, plasma purification steps were done within 30 minutes of collection. The peripheral blood sample was subjected to two centrifugation cycles. The first at 1,400 *g* for 15 minutes for plasma recovery and the second at 3,000 *g* for purification of poor plasma. 20 21 The cfDNA extraction was performed with the Maxwell RSC Extractor, code AS4500, using the Maxwell RSC ccfDNA Plasma Kit, code AS1480 (Promega SRL, Milan, Italy). 22

The amount of extracted cfDNA was measured spectrophotometrically (NanoDrop 2000, Thermo Fisher Scientific Inc, Waltham, United States) and the data were further validated using a capillary electrophoresis system, the 4150 TapeStation System, and Cell-free DNA ScreenTape reagents (Agilent Technologies Inc, Santa Clara, United States). The purity of the extracted material was evaluated by measuring the amount of DNA with a molecular weight between 180 and 200 bp. 23 24

### ddPCR Analysis

ddPCR analysis was performed using the QX200 instrument platform (Bio-Rad Laboratories, Inc, United States). The assays used in this phase of the study are the ddPCR KRAS G12/G13 Screening Kit (#1863506), which consists of a mixture of probes for simultaneous detection of the KRAS variant p.(Gly12Ala) (code dHsaMDV2510586), p.(Gly12Cys) (code dHsaMDV2510584), p.Gly12Asp (code dHsaMDV2510596), p.(Gly12Arg) (code dHsaMDV2510590), p.(Gly12Ser) (code dHsaMDV2510588), p.(Gly12Val) (code dHsaMDV2510592), p.code dHsaMDV2510598), and finally the single assay KRAS p.(Gly12Cys), c.34G > T (code dHsaCP2500584).

In the case of poor cfDNA samples, we performed the analysis in duplicate or triplicate to reach at least 10,000 events and to accurately quantify fractional abundance (corresponding to allele frequency). 25 26

### NGS Material and Methods

NGS analysis was performed using two different instrument platforms and genomic panels. The list of gene targets and the technical specifications are presented in Table 1 . Panel 1 is used for the laboratory diagnostic setting, and is based on a CE-IV-labeled assay, and panel 2 for research use only.

**Table 1:** Technical specifications of the two NGS systems used for ctDNA analysis

	Panel 1	Panel 2
NGS assay	Myriapod NGS Cancer panel DNA, code: NG033 (Diatech Pharmacogenetics SRL, Jesi, Italy)	Oncomine Pan-Cancer Cell-Free Assay, code: A37664 (Thermo Fisher Scientific Inc, Waltham, United States)
Genes list sequenced in hotspot regions	ALK, BRAF, EGFR, ERBB2, FGFR3, HRAS, IDH1, IDH2, KIT, KRAS, MET, NRAS, PDGFRA, PIK3CA, POLE, RET, ROS1	AKT1, ALK, APC, AR, ARAF, BRAF, CHEK2, CTNNB1, DDR2, EGFR, ERBB2, ERBB3, ESR1, FBXW7, FGFR1, FGFR2, FGFR3, FGFR4, FLT3, GNA11, GNAQ, GNAS, HRAS, IDH1, IDH2, KIT, KRAS, MAP2K1, MAP2K2, MET, MTOR, NRAS, NTRK1, NTRK3, PDGFRA, PIK3CA, PTEN, RAF1, RET, ROS1, SF3B1, SMAD4, SMO, TP53
No. of sequenced genes	17	44 a
Certification	CE-IVD	Research Use Only (RUO)
Instrument	MiSeq Dx (Illumina, San Diego, United States)	Ion GeneStudio S5 System (Thermo Fisher Scientific Inc, Waltham, United States)
Analysis tool	Myriapod NGS Data Analysis Software, code: NG900-SW, 5.0.7 version	Ion Reporter Software, 5.18 version

Abbreviations: ctDNA, circulating tumor DNA; NGS, next-generation sequencing.

a Maximum coverage of gene targets for DNA analysis.

The applied analysis criteria for the variant calling are the following:

- Panel 1: “compliant sample” as defined by analysis software; variants over 1% variant allele frequency (VAF) threshold with at least 500x reading.
- Panel 2: “compliant sample” as defined by analysis software; variants over 0.5% VAF threshold with at least 1,000x reading and at least 200 molecular coverage.

## Results

### Liquid Biopsies: Global NGS Results

The first reported datum is the positivity for significant variants from a clinical point of view, classified as pathogenetic or probably pathogenetic or VUS. 27

Of the 39 liquid biopsies analyzed at T0 using panel 1, 7/14 (50%) were positive among the cases of intestinal neoplasia, 7/17 (41%) were positive among lung neoplasms, and 2/8 (25%) were positive among all other cases of malignancies. The same samples analyzed with panel 2 resulted in 9/14 (64%) positive cases among intestinal neoplasms, 7/17 (41%) among lung neoplasms, and 4/8 (50%) among all the other cases of tumors.

The global incidence of positivity obtained considering all positives identified through one or both NGS panels consists of 9/14 (64%) for intestinal neoplasms, 11/17 (65%) for pulmonary, and 5/8 (62%) for all other pathologies. In panel 1, 19/39 (49%) positive cases of the study population were identified and in panel 2 we detected 20/39 (51%) positive cases ( Table 2 ).

**Table 2:** The number of NGS-positive cases and relative incidence in order of sites of tumor origin and type of used panel

	Panel 1	Panel 2	Global positivity incidence for tumor type
**Intestine**	7/14 (50%)	**9/14 (64%)**	9/14 (64%)
**Lung**	7/17 (41%)	7/17 (41%)	**11/17 (65%)**
**Others**	2/8 (25%)	**4/8 (50%)**	5/8 (62%)
**Global positivity incidence for panel type**	16/39 (41%)	20/39 (51%)	

Abbreviations: NGS, next-generation sequencing.

Note: The maximum incidence values per pathology category are shown in bold.

### Liquid Biopsies and Metastatic Pulmonary Cancer: NGS Results

The analysis conducted through panel 1 on the extracted ctDNA from cases of metastatic lung cancer identified 10 variants, 5 of which were exclusive for sequencing gene coverage and 5 communal to panel 2. Panel 2 identified 10 variants, 5 exclusive for sequencing gene coverage and five communal to panel 1. Using two panels allowed identifying five variants commonly covered by the two analysis systems. Every NGS panel allowed the detection of 10 variants, and the combined use of these two platforms identified globally 16 distinct ones ( Table 3 ).

**Table 3:** The number of variants detected on ctDNA of pulmonary neoplasms using both NGS panels

Tumor origin	Lung	Panel 1	Panel2
**NGS panels' common variants,** ***n*** **(%)**	Detected	5 (31%)	5 (31%)
	Not detected	1 (6%)	1 (6%)
**NGS panel–related exclusive variants (** ***n*** **)**	Detected	5 (31%)	5 (31%)
	Total variants detected by each panel	10 (62%)	10 (62%)
	Total detected variants	16 (100%)	

Abbreviations: ctDNA, circulating tumor DNA; NGS, next-generation sequencing.

Note: These are classified considering exclusive and communal ones for panel design.

Analysis conducted with panel 1 revealed five PIK3CA variants, three of which were located in exon 2 (D64G, L94P and K111R) and two in exon 10 (N526D and E545K). Two mutations were detected in exon 18 of the EGFR gene (L704V and E709_T710delinsD).

Finally, it has been possible to reveal globally three variants for the following three genes: NRAS, KRAS, and ERBB2, located in exon 4 (Q99R), exon 2 (G12C), and exon 20 (A775_G776insYVMA), respectively. Panel 2 sequencing revealed three variants in exon 5 (P177_C182del and R156P) and exon 4 (P152L) of the TP53 gene, two in exon 19 (E746_A750delELREA) and exon 18 (E709_T710delinsD) of the EGFR, and only one for the following three genes: PIK3CA, KRAS, and ERBB2, located in exon 10 (E545L), in exon 2 (G12C), and exon 20 (A775_G776insYVMA), respectively. The two systems detected all the variants common to the coverage of the two panels except PIK3CA L94P and E746_A750delELREA ( Table 4 ). There is no statistically significant difference in the allelic frequency measurements of four variants (ERBB2 A775_G776insYVMA, KRAS G12C, PIK3CA E545K, and EGFR E709_T710delinsD) identified by both panels (p.0.4734).

**Table 4:** List of variants detected in the ctDNA of the metastatic pulmonary cases using panels 1 and 2

	Panel 1				Panel 2			
Case no.	NGS result: gene and variant	Variant annotation	VAF (%)	Exon	NGS result: gene and variant	Variant annotation	VAF (%)	Exon
1	No Variant Detected	/	/	/	NVD	/	/	
2	*PIK3CA p.(Asp64Gly)*	VUS	1.53	2	NVD	/	/	
3	NVD	/	/	/	NVD	/	/	
4	**PIK3CA p.(Leu94Pro)**	**LP**	**1.07**	2	NVD	/	/	
5	NVD	*/*	*/*	*/*	**EGFR p.(Glu746_Ala750delELREA)**	**P**	**1.3**	**19**
					*FGFR3 p.Phe384Leu*	LP	51	9
6	NVD	/	/	/	NVD	/	/	
7	**ERBB2 p.(Ala775_Gly776insTyrValMetAla)**	**LP**	**1.4**	20	**ERBB2 p.(Ala775_Gly776insTyrValMetAla)**	**LP**	0.63	20
8	NVD	/	/	/	NVD	/	/	/
9	*PIK3CA p.(Asn526Asp)*	P	1.07	10	NVD	/	/	/
	*EGFR p.(Leu704Val)*	LP	2.13	18				
	*NRAS p.(Gln99Arg)*	VUS	1.12	4				
10	*PIK3CA p.(Lys111Arg)*	LP	1.42	2	NVD	/	/	/
11	NVD	/	/	/	NVD	/	/	/
12	NVD	/	/	/	*TP53 p.(Pro152Leu)*	P	3.8	4
13	**KRAS p.(Gly12Cys)**	**P**	**8.27**	2	**KRAS p.(Gly12Cys)**	**P**	4.6	2
					*TP53 p.(Pro177_Cys182del)*	P	2.9	5
14	NVD	/	/		*TP53 p.(Pro278Thr)*	P	1.5	8
15	**PIK3CA p.(Glu545Lys)**	**P**	**11.07**	10	**PIK3CA p.(Glu545Lys)**	**P**	12.8	10
	**EGFR p.(Glu709_Thr710delinsAsp)**	**P**	**10.04**	18	**EGFR p.(Glu709_Thr710delinsAsp)**	**P**	42.4	18
16	NVD	/	/	/	*TP53 p.(Arg156Pro)*	P	3.4	5
17	NVD	/	/	/	NVD	/	/	

Abbreviations: ctDNA, circulating tumor DNA; LP, likely pathogenic; NGS, next-generation sequencing; P, pathogenic; VAF, variant allele frequency; VUS, variant of uncertain significance.

Note: The variants written in bold are covered by the design of the two panels. The variability, in terms of the inaccuracy of the measurement for VAF value, is 10% (95% confidence interval).

### Liquid Biopsies and Metastatic Intestinal Cancer: NGS Results

The analysis conducted through panel 1 on the extracted ctDNA from cases of metastatic intestinal cancer allowed identifying eight variants; one is exclusive for sequencing gene coverage and seven are communal to panel 2. Panel 2 application made it possible to identify 13 variants, 4 exclusive for sequencing gene coverage, and 9 communal to panel 1. The sequencing performed with panel 2 allowed us to detect a greater absolute number of variants than the panel 1 application (13 vs. 8; Table 5 ).

**Table 5:** The number of variants detected on ctDNA of intestinal neoplasms using both NGS panels

Tumor origin	Intestine	Panel 1	Panel2
**NGS panels' common variants,** ***n*** **(%)**	Detected	7 (47%)	9 (60%)
	Not detected	3 (20%)	1 (7%)
**NGS panel–related exclusive variants,** ***n*** **(%)**	Detected	1 (7%)	4 (27%)
	Total variants detected by each panel	8 (53%)	13 (87%)
	Total detected variants	15 (100%)	

Abbreviations: ctDNA, circulating tumor DNA; NGS, next-generation sequencing.

Note: These are classified considering exclusive and communal ones for panel design.

Analysis conducted with panel 1 revealed four KRAS variants, three of which are located in exon 2 (G12C, G12D, and G12A) and one in exon 3 (Q61L). Two mutations were detected in exon 5 of PIK3CA (N345K and N350G) and only two variants resulted in exon 15 of the BRAF gene (K601E and V600E). Panel 2 sequencing revealed 5 variants in exon 2 (G13D, G12D, G12C, and G12A) and exon 3 (Q61L) of the KRAS gene, 1 each in exon 5 (N345K) and exon 9 (E545K) of PIK3CA, and only 1 for the following three genes, SF3B1, APC, and BRAF, respectively, located in exon 15 (K700E), in exon 15 (R1450Ter), and only 1 for the following three genes, SF3B1, APC, and BRAF, all located in exons 15 and are respectively: K700E, R1450Ter, and V600E. Most of the variants common to the coverage of the two panels were detected by the two systems except in two (G13D and G12D) KRAS-positive samples, one K601E BRAF, and one E545K PIK3CA ( Table 6 ). There is no statistically significant difference in the allelic frequency measurements of the six variants (KRAS G12C, G12D, Q61L, G12A, PIK3CA N345K, and BRAF V600E) identified in both panels (p. 0.9150).

**Table 6:** List of the variants detected in the ctDNA of the metastatic intestinal cases using panels 1 and 2

	Panel 1					Panel 2			
Case no.	NGS result: gene and variant	Variant annotation	VAF (%)	Exon	NGS result: gene and variant	Variant annotation	VAF (%)	Exon
1	NVD	/	/	/	NVD	/	/	/
2	NVD	/	/	/	**KRAS p.(Gly13Asp)**	**P**	**0.89**	**2**
				/	TP53 p.(Arg175His)	P	1.2	4
3	NVD	/	/	/	**KRAS p.(Gly12Asp)**	**P**	**1.2**	**2**
4	**KRAS p.(Gly12Cys)**	**P**	**6.92**	**2**	**KRAS p.(Gly12Cys)**	**P**	**0.85**	**2**
					TP53 p.(Arg175His)	P	1.6	4
5	**BRAF p.(Lys601Glu)**	**P**	**1.11**	**15**	SF3B1 p.(Lys700Glu)	P	3.5	15
6	**PIK3CA Asn345Lys**	**P**	**1.19**	5	**PIK3CA p.(Asn345Lys)**	**P**	**16.67**	**5**
	**KRAS p.(Gly12Asp)**	**P**	**3.41**	2	**KRAS p.(Gly12Asp)**	**P**	**3.9**	**2**
7	PIK3CA Asp350Gly	VUS	1.19	5	**PIK3CA p.Glu545Lys)**	**P**	**0.89**	**9**
8	NVD	/	/	/	NVD	/	/	/
9	NVD	/	/	/	NVD	/	/	/
10	NVD	/	/	/	NVD	/	/	/
11	**KRAS p.(Gln61Leu)**	**P**	**2.0**	3	**KRAS p.(Gln61Leu)**	**P**	**3.12**	3
12	NVD	/	/	/	NVD	/	/	/
13	**KRAS p.(Gly12Ala)**	**P**	**28.79**	2	**KRAS p.(Gly12Ala)**	**P**	**20.8**	2
14	**BRAF p.(Val600Glu)**	**P**	**6.7**	15	**BRAF p.(Val600Glu)**	**P**	**7.2**	15
					APC p.(Arg1450Ter)	P	11.2	15

Abbreviations: ctDNA, circulating tumor DNA; LP: likely pathogenic; P, pathogenic; VAF, variant allele frequency; VUS, variant of uncertain significance.

Note: The variants written in bold are covered by the design of the two panels. The variability, in terms of the inaccuracy of the measurement for VAF value, is 10% (95% confidence interval).

### Liquid Biopsies and Other Cancers (Gastric, Mammary, Cholangiocarcinoma, and Melanoma): NGS Results

The analysis conducted through panel 1 on the extracted ctDNA from cases of various cancers allowed identifying two communals covering panel 2 variants. These common coverage variants (CCVs) are detected by NGS panel 1, and only one of these is revealed by NGS panel 2. On the other hand, this last panel allows the detection of three exclusive coverage variants. The total genomic alterations detected by two NGS systems are two for panel 1 and four for panel 2 ( Table 7 ).

**Table 7:** The number of variants detected on ctDNA of other various neoplasms using both NGS panels

Tumor origin	Various (stomach, breast, biliary tract, skin)	Panel 1	Panel 2
**NGS panels' common variants,** ***n*** **(%)**	Detected	2 (100%)	1 (50%)
	Not detected	0 (0%)	1 (50%)
**NGS panel-related exclusive variants (** ***n*** **)**	Detected	0	3
	Total variants detected	2	4

Abbreviations: ctDNA, circulating tumor DNA; NGS, next-generation sequencing.

Note: These are classified considering exclusive and communal ones for panel design.

The use of panel 1 allowed the detection of two common-design variants. These mutations are classified as pathogenic and are located in exon 10 of the PIK3CA gene (E545K) and exon 15 of BRAF (V600E). Panel 2 revealed only one of the common-design variants, which is located in exon 10 of the PIK3CA gene (E545K); nevertheless, this analytical application highlighted three variants of exclusive coverage, one classified as probably pathogenetic, present in exon 4 of the FGFR3 (F384L), and two as pathogenetic in exon 8 of the ESR1 gene (Y537N) and exon 4 of the TP53 gene (R175H; Table 8 ). A sufficiently high number was not reached to compare the statistical significance between the VAF values of the common variants detected by the two panels.

**Table 8:** List of variants detected in the ctDNA from cancer patients with various tumor origins (stomach, breast, biliary tract, skin) using panels 1 and 2

		Panel 1				Panel 2			
Case no.	Localization	NGS result: gene and variant	Variant annotation	VAF (%)	Exon	NGS result: gene and variant	Variant annotation	VAF (%)	Exon
1	Breast	NVD	/	/		FGFR3 p.Phe384Leu)	LP	57.9	4
2	Breast	NVD	/	/		ESR1 p.Tyr537Asn)	P	1.8	8
3	Breast	**PIK3CA p.(Glu545Lys)**	**P**	**4.3**	**10**	**PIK3CA p.(Glu545Lys)**	**P**	**2.6**	10
1	Skin	**BRAF p.(Val600Glu)**	**P**	**1.7**	**15**	NVD	/	/	/
1	Stomach	NVD	/	/	/	TP53 p.(Arg175His)	P	11.7	4
2	Stomach	NVD	/	/	/	NVD	/	/	/
3	Stomach	NVD	/	/	/	NVD	/	/	/
1	Biliary tract	NVD	/	/	/	NVD	/	/	/

Abbreviations: ctDNA, circulating tumor DNA; LP, likely pathogenic; NGS, next-generation sequencing; P, pathogenic; VAF, variant allele frequency.

Note: The variants written in bold are covered by the design of the two panels. The variability, in terms of the inaccuracy of the measurement for VAF value, is 10% (95% confidence interval).

### Comparison NGS Results

**Fig. 3:**
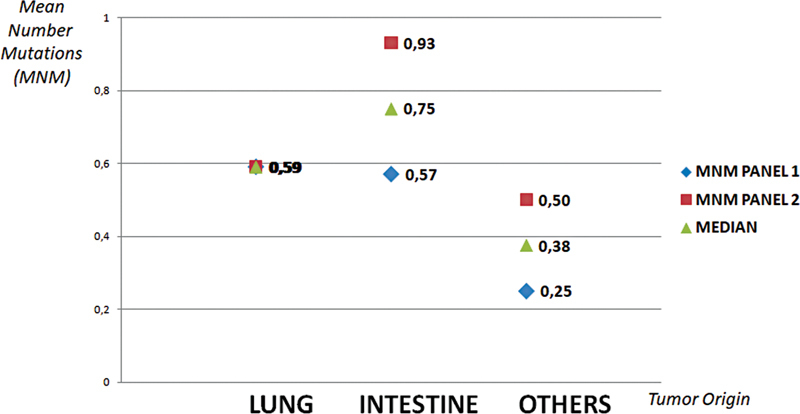
The mean number of mutations among the three cancer population cohorts (intestine, lung, and another origin). These values are calculated for every next-generation sequencing (NGS) panel type.

The study population has been analyzed considering subdivision into pulmonary, intestinal, and other pathologies ( Fig. 3 ). The mean number of mutations (MNM) was evaluated and is defined by the total number of mutations identified in these cohorts divided by the number of cases analyzed. The median MNM is higher among intestinal cases ( *n* = 0.78) than pulmonary ( *n* = 0.67) and other origin ( *n* = 0.44) cases. The median MNM values are higher for the intestine setting (0.75) and lower for lung and other neoplasms (0.59 and 0.38, respectively).

CCVs have been detected using two different panels. We have found 15 by panel 2 use and 14 by panel 1 ( Table 9 ). Most of the CCVs detected by panel 1 are confirmed by panel 2 (79%) and the mean VAF has been higher for panel 2 findings than those for panel 1 (11.22 vs. 6%). Both panels detect the same number of PIK3CA and ERBB2 variants. Different quotes are revealed for KRAS ( *n* = 7 and 5 using, respectively, panels 2 and 1), EGFR ( *n* = 2 and 1 for panels 2 and 1, respectively), and for BRAF ( *n* = 3 for panel 1 and n = 1 for panel 2).

**Table 9:** The number of common-design variants detected by two NGS panels, classified for gene type, mean VAF, and concordance performance

				No. of common variant/s detected per gene					
	Common variants identified ( *n* )	Confirmed by the other NGS panel ( *n* )	Mean VAF common variants (%)	PIK3CA	ERBB2	KRAS	EGFR	BRAF	Common variants globally identified ( *n* )
**Panel 1**	14	11 (79%)	6.00	4	1	5	1	3	17
**Panel 2**	15	11 (73%)	11.22	4	1	7	2	1	

Abbreviations: cfDNA, circulating free DNA; NGS, next-generation sequencing; VAF, variant allele frequency.

### Data Comparison between Liquid Biopsy and Tissue Analysis

For 13 patients enrolled in the study, it was possible to compare the data obtained with NGS analyses on plasma and tissue material with nonsynchronous sampling times. These are eight cases of intestinal neoplasia, four pulmonary, and one melanoma. For seven patients of this cohort, we have a complete agreement (CA) analytical result (five intestinal, one pulmonary, and one melanoma case). There is a partial agreement (PA) sequencing for two patients (intestinal and pulmonary).

In an intestinal case, KRAS Q61L variant was detected in both plasma and tissue, but only in tissue has it been possible to detect PIK3CA mutation (G545L). The other PA result is a pulmonary case where the ERBB2 A775_G776insTVMA variant was detected in both samples, but the PIK3CA K111N mutation was found only in tissue. The discordant results (D) were found in four cases, two in intestinal and two in pulmonary cases. The latter were negative in plasma, one tissue was positive for the NRAS A59T variant and the other was positive for KRAS G12C mutation. One of the two intestinal discordant results was discordant for PIK3CA variants (D350G in plasma and G545K in tissue) and for detecting KRAS G12D mutation only in the tissue sample. The 1 INT case was negative in the ctDNA sample but positive for NRAS Q61K and KRAS A59E in the tissue matrix ( Table 10 ).

**Table 10:** NGS results obtained by panel 1 on plasma and tissue of enrolled patients

cfDNA analysis							Tissue analysis			
Case	cfDNA conc. (pg/µL)	cfDNA purity (%)	ΔT LB (d)	Variant	Variant type	VAF (%)	Variant	Variant type	VAF (%)	Agreement
12 Int	43	79	6	No variant detected	/	/	No variant detected	/	/	CA
13 Int	22.9	87	–13	KRAS p.(Gly12Ala)	P	28.8	KRAS p.(Gly12Ala)	P	37.0	CA
14 Int	21.4	100	362	BRAF p.(Val600Glu)	P	6.7	BRAF p.(Val600Glu)	P	19.8	CA
15 Lung	162	72	14	EGFR p. (Glu709_Thr710delinsAsp)	P	10.0	EGFR p.(Glu709_Thr710delinsAsp)	P	15.0	CA
				PIK3CA p.(Glu545Lys)	P	11.1	PIK3CA p.(Glu545Lys)	P	4.0	
1 Mel	24.6	75	–21	BRAF p.(Val600Glu)	P	1.7	BRAF p.(Val600Glu)	P	2.2	CA
4 Int	35.7	73.52	–22	KRAS p.(Gly12Cys)	P	6.9	KRAS p.(Gly12Cys)	P	27.0	CA
10 Int	43	79	15	No variant detected	/	/	NO VARIANT DETECTED	/	/	CA
11 Int	285	84.41	15	KRAS p.(Gln61Leu)	P	2.0	KRAS p.(Gln61Leu)	P	9.3	PA
							PIK3CA p.(Glu545Lys)	P	3.6	
7 Lung	50.9	74	–111	ERBB2 p.(Ala775_Gly776insTyrValMetAla)	LP	1.4	ERBB2 p.(Ala775_Gly776insTyrValMetAla)	LP	23.0	PA
							PIK3CA 2. p.(Lys111Asn)	LP	10.0	
7 Int	71.9	80.14	112	PIK3CA p.(Asp350Gly)	VUS	1.2	PIK3CA p.(Glu545Lys)	P	24.4	D
							KRAS p.(Gly12Asp)	P	23.5	
1 Int	25.3	82.01	–7	No variant detected	/	/	NRAS p.(Gln61Lys)	VUS	10.5	D
							KRAS p.(Ala59Glu)	P	10.7	
11 Lung	17	78.9	14	No variant detected	/	/	NRAS p.(Ala59Thr)	LP	21.5	D
16 Lung	84.9	72.15	928	No variant detected	/	/	KRAS p.(Gly12Cys)	P	51.0	D

Abbreviations: CA, complete agreement; cfDNA, circulating free DNA; D, discordance; LP, likely pathogenic; NGS, next-generation sequencing; P, pathogenic; PA, partial agreement; VAF, variant allele frequency; VUS, variant of uncertain significance.

Note: The table shows the dosages of cfDNA in picograms per microliter (cfDNA conc.), the purity in percentage (cfDNA purity), the time difference expressed in days by the liquid biopsy execution to the tissue collecting (DT LB), the variants detected with the VAF and their classification. These data are reported for plasma and tissue. The agreement estimates of the results relating to the single variants are also reported. The variability, in terms of the inaccuracy of the measurement for VAF value, is 10% (95% confidence interval) and that for other values is 5% (95% confidence interval).

### Monitoring Data

The yield of cfDNA extraction was evaluated (see section Methods). The cfDNA mean (ng/mL) has been considered and registered by tumor origin and collection times (T0–T10). At the cfDNA, the mean is highest for pulmonary pathologies (976.8) than for intestinal and other pathologies (306.5 and 550.7, respectively). There is no statistically significant difference in the cfDNA mean among the three patient groups ( *p* = 0.21). For this time of collection, the cfDNA median value is higher in the “other” neoplasms (263.5) than intestinal (182.0) and pulmonary (167.0) neoplasms. During this preliminary phase of the study, the greatest monitoring sample collection has been realized for the intestinal pathologies ( *n* = 55) followed by the pulmonary ( *n* = 46) and other pathologies ( *n* = 16; Table 11 ).

**Table 11:** The mean and median cfDNA dosage values and the number of collected samples were subdivided by tumor origin (intestinal, pulmonary, and other sites) and recorded for the various monitoring times (T0–T10)

		T0	T1	T2	T3	T4	T5	T6	T7	T8	T9	T10
cfDNA mean a (ng/mL)	Lung	976.8	157.0	193.3	275.0	248.6	186.0	170.0	/	/	/	/
	Intestine	306.5	299.0	693.4	537.2	1,562.6	293.2	193.7	300.0	506.0	1,115.5	250.0
	Others	550.6	96.6	286.0	228	218	211	/	416	/	/	/
Samples ( *n* )	Lung	17	5	8	3	5	3	2	3	0	0	0
	Intestine	14	7	5	6	5	4	4	3	3	3	3
	Others	8	3	1	1	1	1	0	1	0	0	0
cfDNA median a (ng/mL)	Lung	167.0	170.0	194.0	275	265.0	186.0	170.0	/	/	/	/
	Intestine	182.0	237.0	294.0	245.5	213.0	284.0	212.0	300.0	506.0	1,115.5	289.0
	Others	263.5	109.0	286.0	228.0	218.0	211.0	/	416.0	/	/	/

Abbreviations: cfDNS, circulating free DNA.

a The variability, in terms of the inaccuracy of the measurement for cfDNA mean and median values, is 5% (95% confidence interval).

In this phase of the study, it has been possible to monitor four cases of KRAS-positive intestinal neoplasia for which at least five samples had already been collected (patient 2 INT with G13D variant, 3 INT, and 6 INT with G12D and case 4 INT with G12C). In the 6 INT case, we performed nine monitoring points (T0–T9), and the patient was lost at T10 due to a cardiovascular stroke. The 4 INT patient has not complied with the collection of samples at T4 and T7, the 2 INT rejected T4 and T7 biopsies, and 3 INT was lost at T5 point. In every collection, plasmatic cfDNA levels (ng/mL) and VAF (%) have been measured, and all these data are reported in Table 12 .

**Table 12:** Monitoring kinetics for four cases of intestinal neoplasia (6 Int in section A, 4 Int in B, 2 Int in C, and 3 Int in D)

**(A)**	**Case: 6 INT**											
	Timing	T0	T1	T2	T3	T4	T5	T6	T7	T8	T9	T10
	LB VAF (%)	3 ± 0.1	2.5 ± 0.1	1.6 ± 0.1	5.6 ± 0.3	0.5 ± 0.0	0.0	1	1.3 ± 0.1	1.8 ± 0.1	3.7 ± 0.2	/
	cf DNA (ng/mL)	249 ± 12.4	727 ± 36.3	298 ± 15	174 ± 8.7	213 ± 10.7	284 ± 14.2	212 ± 10.6	452 ± 22.6	593 ± 29.7	1,650 ± 82.5	/
	Clinical outcome	/	PR	PD	PD	PD	PD	PD	SD	SD	SD	Lost
**(B)**	**Case: 4 INT**											
	Timing	T0	T1	T2	T3	T4	T5	T6	T7	T8	T9	T10
	LB VAF (%)	7 ± 0.3	0.6 ± 0.0	2.2 ± 0.1	1.2 ± 0.1	0.6 ± 0.0	0.0	0.2 ± 0.0	Missing	Missing	Missing	0.0
	cf DNA (ng/mL)	73.52 ± 3.7	233 ± 11.7	290 ± 14.5	140 ± 7	67 ± 3.3	442 ± 22.1	155 ± 7.7				289 ± 14.4
	Clinical outcome	/	PR	PR	PR	PR	SD	SD	SD	SD	SD	SD
**(C)**	**Case: 2 INT**											
	Timing	T0	T1	T2	T3	T4	T5	T6	T7	T8	T9	T10
	LB VAF (%)	2 ± 0.1	0.9 ± 0.5	0.0	0.3 ± 0.2	Missing	0.0	2.3 ± 0.1	Missing	0.15 ± 0.0	0.0	0 ± 0
	cf DNA (ng/mL)	182 ± 9.1	241 ± 12.0	237 ± 12	317 ± 16		261 ± 13	238 ± 12		419 ± 21	581 ± 29	380 ± 19
	Clinical outcome	/	SD	SD	SD	SD	SD	SD	SD	SD	PD	PD
**(D)**	**Case: 3 INT**											
	Timing	T0	T1	T2	T3	T4	T5	T6	T7	T8	T9	T10
	LB VAF (%)	1 ± 0.0	43 ± 2.2	51.1 ± 2.6	63.7 ± 3.2	43 ± 2.2	Missing	/	/	/	/	/
	cf DNA (ng/mL)	25.3 ± 1.3	610 ± 30.5	2,450 ± 122.5	1,780 ± 89	5,720 ± 286		/	/	/	/	/
	Clinical outcome	/	PD	PD	PD	PD	Lost	/	/	/	/	/

Abbreviations: cfDNS, circulating free DNA; Lost, death; Missing, the patient did not reach the visit; PD, progression disease; PR, partial response; SD, stable disease.

Note: For each case, results corresponding to every blood draw are reported: cfDNA levels, variant allele frequency (VAF) measured by droplet digital polymerase chain reaction (ddPCR) assay, and clinical outcome based on radiological response and general patient condition.

## Discussion

The number of variants found through NGS technology, applied to the three patient populations (lung, intestinal, and other neoplasms), was globally higher for pulmonary than for others ( Table 2 ). The two NGS panels seem equally efficient in cases of lung cancers ( Fig. 3 ), and this point is probably due to a similar coverage design for pulmonary target-therapy variants, a crucial aspect of every commercial NGS panel. 28 We found some differences in the number of variants identified by the two panels used. Panel 2 seems more effective in the intestinal setting, probably because the higher number of sequenced genes than panel 1 allows us to realize a more efficient investigation of these genomically unstable pathologies. This consideration is also supported by data on detecting the NGS panel–related exclusive variants. The number of these variants detected by panel 2 is greater than the ones found by panel 1 in intestinal cases (4 vs. 1) and other malignancies (3 vs. 0), but there are no quantitative differences in the lung cases (see Tables 3 , 5 , and 7 ).

**Fig. 4:**
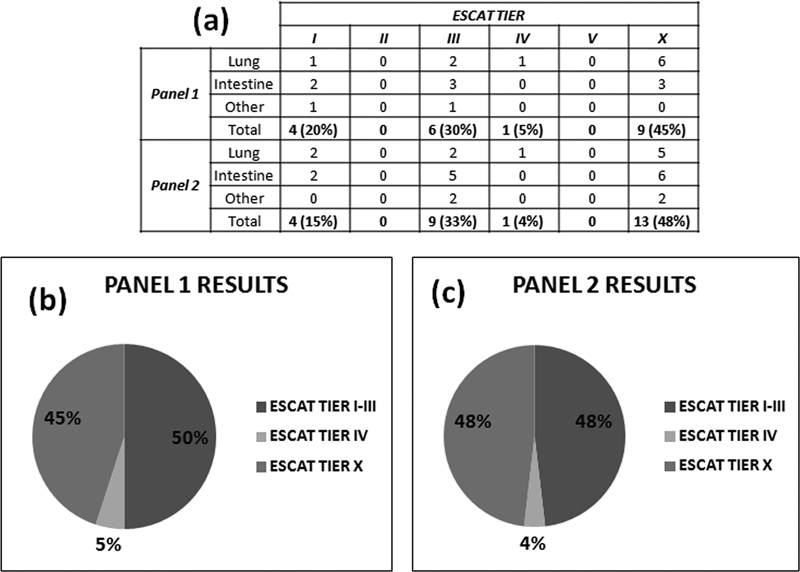
ESMO Scale for Clinical Actionability of molecular Targets (ESCAT) tiers of the identified mutations in the plasma of patient cohort ( *n* = 39). ( **a** ) The results are classified according to the NGS panel results. All the mutations are included among I to IV and X ESCAT tiers. ( **b,c** ) The pie charts show the percentual incidence of these variant types by each NGS panel result.

All the variants identified in ctDNA by NGS methods can be classified on the basis of the ESMO Scale for Clinical Actionability of molecular Targets (ESCAT) actionability. 29 No significant differences emerged with the use of the two NGS panels as regard the operability of the identified variants. These data confirm the clinical impact value of the use of small low-throughput panels (<50 genes) such as panel 1. 30 Most of the identified mutations lack evidence for actionability (X tier). On the other hand, tiers I to III constitute half of the observed molecular alterations ( Fig. 4 ). These last data confirm significant liquid biopsy value as a tool to identify high clinical valence molecular data.

The validity of the two used NGS panels in the analysis of liquid biopsies can be further appreciated by considering the number of cases for which there was complete molecular response agreement (CA) versus those for which there was only partial (PA) or discordance (D) agreement. The percentage of CA cases is 51% ( *n* = 20), and this rises to 59% ( *n* = 20 + 3) if we consider the real difference in molecular data among the PA ones ( Table 13A ). The PA cases are one pulmonary and two intestinal. In this set, panel 2 allowed the detection of further variants in addition to the druggable KRAS and BRAF mutations, two TP53, and one APC ( Table 13B ). These additional alterations did not impact therapeutic decision-making compared to KRAS and BRAF events. The global molecular response for these PA cases had the same clinical value as the other CA cases.

**Table 13:** Laboratory molecular response using both NGS panels

(a)		Laboratory response on individual cases								
		Lung		Intestine			Others		Entire cohort	
Complete agreement		8 (47%)		8 (57%)			4 (50%)		20 (51%)	
Partial agreement		1 (6%)		2 (14%)			0 (0%)		3 (8%)	
Discordance		8 (47%)		4 (29%)			4 (50%)		17 (41%)	
Total cases		17 (100%)		14 (100%)			8 (100%)		39 (100%)	
**(b)**		**Panel 1**					**Panel 2**			
**Case no.**	**Localization**	**NGS result**	**NGS annotation**	**NGS VAF (%)**	**Exon**	**NGS result**	**NGS annotation**	**NGS VAF (%)**	**Exon**
13	Lung	KRAS p.(Gly12Cys)	Pathogenic	8.27	2	KRAS p.(Gly12Cys)	Pathogenic	4.6	2
						**TP53 p.(Pro177_Cys182del)**	**Pathogenic**	**2.9**	**5**
4	Intestine	KRAS p.(Gly12Cys)	Pathogenic	6.92	2	KRAS p.(Gly12Cys)	Pathogenic	0.85	2
						**TP53 p.(Arg175His)**	**Pathogenic**	**1.6**	**4**
14	Intestine	BRAF p.(Val600Glu)	Pathogenic	6.7	15	BRAF p.(Val600Glu)	Pathogenic	7.2	15
						**APC p.(Arg1450Ter)**	**Pathogenic**	**11.2**	**15**

Abbreviations: NGS, next-generation sequencing; VAF, variant allele frequency.

Note: (a) The concordant NGS results are classified as complete agreement (CA), while those with at least one variant commonly detected by both panels are considered partial agreement (PA), and different molecular profiles are classified as discordant (D). (b) The PA cases differ for TP53 and APC variants and present druggable mutations (KRAS and BRAF) detected by both panels (results in *bold* in Table 13b ).

The study made it possible to compare the predictivity of the liquid biopsy by comparing the NGS result (panel 1) of 13 tissue samples with the respective circulating DNA samples ( Table 10 ). Considering this subset, we realize that the ctDNA investigation can confirm tissue data (7/13 samples, 54%). The PA cases (2/13, 15%) have in common the lack of detection on the plasma of minority (evidently subclonal) variants. The (D) cases demonstrate that liquid biopsy should be used as a complementary and not an alternative tool. The discordance data incidence (4/31, 31%) confirmed previous studies investigating this topic. 31

**Fig. 5:**
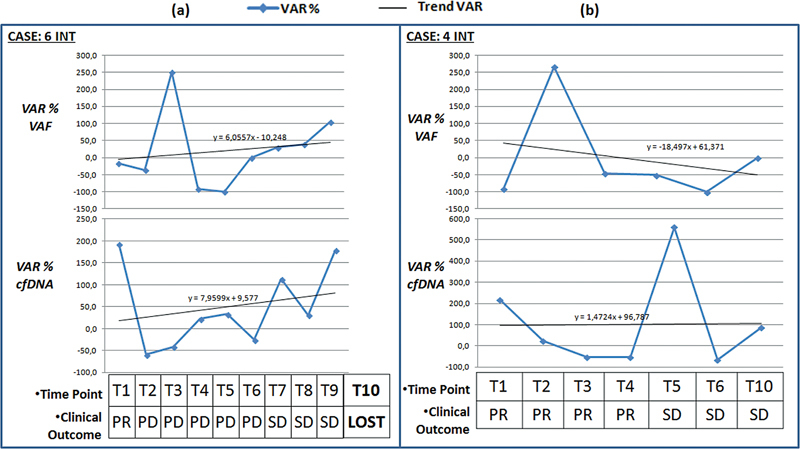
The graphics of the two sections show the trend of the molecular response in terms of percentage change in variant allele frequency (VAF) compared to the previous measurement (VAR% VAF) starting from T1 for ( **a** ) case 6 INT and ( **b** ) case 4 INT. The graphics at the bottom of the two sections show the trend of the molecular response in terms of percentage change in the levels of cfDNA compared to the previous measurement (VAR% cfDNA). At the various monitoring points, the global clinical status of the patient is indicated. LOST, patient's death; PR: partial response; PD, disease progression; SD, stable disease.

**Fig. 6:**
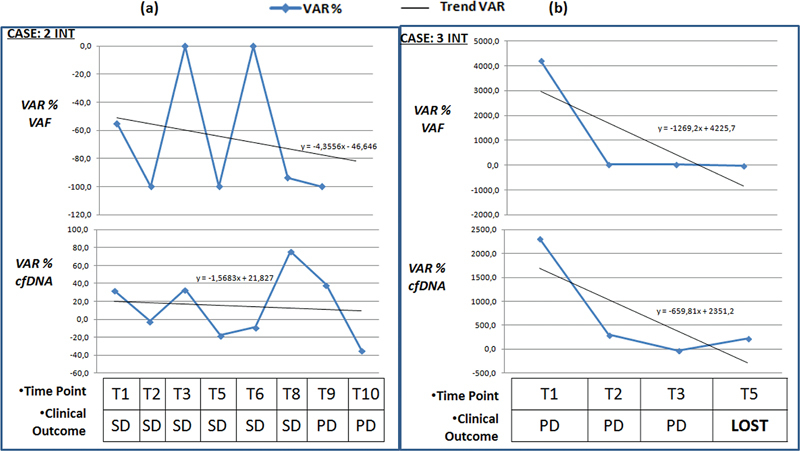
The graphics above of the two sections show the VAR % VAF starting from T1 for case 2INT ( **a** ) and case 3INT ( **b** ). The graphics at the bottom of the two sections show VAR % cfDNA. At the various monitoring points, the global clinical status of the patient is indicated. cfDNA, circulating free DNA; LOST, patient's death; PD, disease progression; SD, stable disease; VAR. variant).

Regarding the ctDNA level analysis, we could conclude that there are no statistically significant differences among the groups of pathologies and monitoring kinetic points. The data confirm that the levels of circulating DNA cannot be considered individually valid as a disease monitoring tool. 19 Nevertheless, the plasmatic recorded fluctuations can be elaborated with other molecular parameters as the VAF of the single variants detected in the tumor genotype. Considering the intestinal cases that carried out at least five monitoring points (the pulmonary setting had low follow-up compliance due to disease lethality or poor patient openness to come to the hospital during the COVID period), suggestive data emerge by comparing the ctDNA levels and the VAF with disease course based on radio diagnostic data and clinical signs. It is known that the plasma levels of circulating DNA are strongly influenced by a series of factors, such as the patient's age, therapy, preanalytical variables, and clinical status (e.g., inflammation, infections, etc.). Trying to reduce these confounding effects, we evaluated the percentage change in the amount of ctDNA compared to the previous sampling (VAR % cfDNA). Considering this parameter with the percentage variation of the VAF of the specific variant identified (VAR % VAF) compared to the previous sampling, a trend emerges that can be associated with the progress of disease ( Figs. 5 and 6 ). The 6 INT case analysis (KRAS G13D positive) revealed a significant increasing trend for the VAR % VAF and VAR % cfDNA. This trend is followed by a negative clinical progression culminating in the patient's death at the 10th month of monitoring ( Fig. 5a ). Instead, for the 4 INT case (KRAS G12C positive), a clear decreasing trend of the VAR % VAF and the VAR % cfDNA is observed. For this patient, there is a partial response (PR) up to disease stability (SD; Fig. 5b ).

Regarding the 2 INT case (KRAS G13D positive), the trend for the VAR % VAF is decreasing and the VAR % cfDNA decrease is not so clear. The allelic frequency levels in the circulation are relatively low, but the cfDNA levels are decidedly high compared to the normal population ( Table 12C ). From a clinical point of view, the disease remains stable for six monitoring cycles, and disease progression is recorded in the T9 and T10 phases. For this patient, it can be concluded that the levels of the circulating mutated clone and the overall cfDNA load do not have a homogeneous trend. The result appears to be slow growth after a relatively long period of stable disease ( Fig. 6a ).

Case 3 INT (KRAS G12D positive) has a decreasing trend for both VAR % VAF and VAR % cfDNA. Nevertheless, the absolute levels of T1–T4 cfDNA are markedly high (from 610 to 5,720 ng/mL), and the allele frequency of the circulating variant is always higher than 40% ( Table 12D ). The consistent presence of circulating neoplastic clones probably determined the molecular response, even if the data should be considered a sign of advanced disease status. The patient died during the fifth month of monitoring ( Fig. 6b ).

The broad inclusiveness of this study could be considered a bias to the interpretation of the results. On the other hand, this data heterogeneity could be fit for understanding the complexity of the patient cohort typically managed by the oncology sector. Most studies on liquid biopsy enroll patients at the time of diagnosis and those with early-stage disease, but confounding effects can arise precisely in these samples. Analysis of cfDNA and associated gene sequencing is an area that plausibly has a different sensitivity than the radiological staging system. The same patient staged for the early-stage disease may already be in a relatively advanced molecular stage. This aspect is valid for the patient in complete remission or PR. Studying patients with different degrees of disease allows us to distinguish several molecular characteristics and limitations of the analytical methods among the various groups.

## Conclusions

Comparison of different NGS panels in this study has currently highlighted that even small-size panels (<50 genes) can be considered highly reliable, especially for the pulmonary setting. Unfortunately, use of medium or small-size NGS panels is not as effective in identifying targets for therapeutic purposes in neoplasms such as the biliary, gastric, and urothelial tract neoplasms.

The BL-MOL-AR study confirmed the relatively high concordance of analytical results between liquid biopsy and tissue analysis (about 60% of cases). The rest of the unconfirmed cases lead us to recommend LB as an additional rather than as an alternative tool for tissue investigation.

Although these data from the BL-MOL-AR study are preliminary and further studies will be necessary, especially for lung pathologies and other “nonintestinal” neoplasms, some conclusions can be drawn on the importance of liquid biopsy. The difficulty of obtaining tissue sample has led to the wide acceptance of the use plasma as an analysis matrix in pulmonary neoplasms. In pulmonary neoplasms, the NGS panels allow us to investigate multiple targets beyond the EGFR and to increase analytical predictivity and effectiveness. On the other hand, ddPCR is fit for monitoring. This technique can provide suggestive information about disease progression, but only if it is correlated with the quantification of the cfDNA levels. This study confirmed that liquid biopsy data should be exclusively interpreted with other information. The cfDNA analysis probably will be able to add prognostic information and disease progression together with data obtained from clinical case management practices such as radiodiagnostic and clinical signs of the patient. It is necessary to encourage studies researching the importance of liquid biopsy in cancer patients to determine the appropriate use of this tool.
